# Combining Ability Analysis and Identification of Testers for Biomass Yield and Forage Quality Traits in Guineagrass (*Megathyrsus maximus*)

**DOI:** 10.3390/plants15172625

**Published:** 2026-08-28

**Authors:** Luis M. Hernandez, Carlos A. Acuña, Rosa N. Jauregui

**Affiliations:** 1 Alliance Bioversity & CIAT, Palmira 763537, Colombia; r.jauregui@cgiar.org; 2 Grupo de Genética y Mejoramiento de Especies Forrajeras, Instituto de Botánica del Nordeste (IBONE, CO-NICET-UNNE), Facultad de Ciencias Agrarias, Universidad Nacional del Nordeste (FCA-UNNE), Corrientes 3400, Argentina; caalac77@gmail.com

**Keywords:** guineagrass, combining ability, apomixis, Panicum, tester selection, tropical forage breeding

## Abstract

Guineagrass (*Megathyrsus maximus*), a key tropical forage species, presents breeding constraints due to apomixis, polyploidy and a narrow sexual gene pool. This study quantified the genetic architecture of biomass and forage quality traits and identified apomictic testers using an incomplete North Carolina II mating design. A total of 175 F_1_ families derived from 27 sexual female parents and nine apomictic male parents were evaluated. Agronomic traits were evaluated across three contrasting environments in Colombia, whereas nutritional quality traits were assessed in one location. Mixed models were used to estimate variance components, combining ability, heritability, and genetic interactions with locations. General combining ability accounted for 58.6% to 80.1% of the main genetic variance depending on the trait. Full-sib family mean broad-sense heritability was high for agronomic traits (*H*^2^*_FS_* = 0.89–0.95) and moderate for nutritional quality traits (*H*^2^*_FS_* = 0.56–0.66), supporting selection at the family level. Male half-sib family mean heritability was consistently high, indicating reliable estimation of apomictic tester effects. Mombaça (CIAT_6962) was the most consistent productivity tester, whereas CIAT_6799 and CIAT_6986 showed favorable predicted effects for nutritional quality traits under Palmira Location. These results provide a quantitative basis for recurrent selection using testers and family validation in guineagrass breeding.

## 1. Introduction

Guineagrass (*Megathyrsus maximus* (Jacq.) B.K. Simón & S.W.L. Jacobs) is one of the most important tropical forage grasses because of its high biomass production, leafiness, persistence, and nutritive value [1,2]. These attributes have supported its widespread use in livestock production systems across Africa, Latin America, and Asia. Beyond its forage value, guineagrass also has ecological relevance due to its C4 photosynthetic pathway, extensive root system, and biological nitrification inhibition (BNI), which may contribute to improved nitrogen cycling, greenhouse gas mitigation, and carbon sequestration in tropical agroecosystems [3,4,5]. However, guineagrass has a dual biological role: while it is a valuable forage resource in managed production systems, it may also behave as an invasive grass in some ecosystems [2]. Therefore, genetic improvement efforts should focus on developing well-characterized cultivars with high productivity, persistence, and forage quality, adapted to specific target environments.

Despite its high agronomic and ecological value, the genetic improvement of this species presents different intrinsic biological constraints. The species is predominantly apomictic [6], reproducing asexually through aposporous gametophytic apomixis, which limits meiotic recombination and the generation of novel genetic variation [7,8]. Moreover, guineagrass is an autotetraploid species (2n = 4x = 32), with cytological studies reporting chromosomal number variation and ploidy variation [9,10], and some reviews highlighting the genomic complexity associated with polyploid tropical forages [11,12]. Although sexual diploid cytotypes are present in natural populations, they are rare and constitute a very narrow genetic base for breeding [8]. Consequently, cultivated materials often present limited genetic diversity [13], which reinforces the need to broaden the breeding base using conserved germplasm and synthetic sexual populations.

The success of plant breeding programs largely depends on the choice of the breeding method, which is determined, in part, by the reproductive biology of the species. Similar to the *Urochloa* breeding program conducted by the Alliance Bioversity International and CIAT, and to maximize the use of apomixis while increasing heterotic effects in sexual populations through successive breeding cycles, a model scheme for improving apomictic species would be recurrent selection based on specific combining ability “RS-SCA” [14]. The RS-SCA strategy implemented in *Urochloa* comprises three steps: (i) a population of sexual plants is crossed with a single apomictic genotype (tester); (ii) the best performing sexual parents are selected based on progeny performance; and (iii) the selected sexual parents are recombined to form the base population for the next cycle [15]. Therefore, to initiate an RS-SCA scheme in *M. maximus*, two prerequisites are needed: (i) the development of a synthetic sexual population and (ii) the identification of an appropriate apomictic tester. The first prerequisite was completed by crossing a sexual genotype (obtained from USDA GeneBank and identified as PI 570664) with 93 apomictic genotypes from CIAT GeneBank [16]. The next step is to identify the appropriate apomictic tester to be used as a pollen donor and to define an effective breeding strategy for this species.

To overcome the genetic and reproductive barriers and to select an appropriate apomictic tester, it is crucial to integrate quantitative genetic tools to explore genetic variance and identify superior parental combinations for hybridization [17,18]. Combining ability analysis provides a quantitative framework to evaluate the average performance of parents across crosses and the performance of specific parental combinations and allows understanding of the nature of gene action in breeding populations [17,18,19]. GCA captures the effect of additive genes (representing the average performance of a parent across multiple crosses), whereas SCA captures the effect of non-additive genes, including dominance and epistasis, that cause certain hybrid combinations to perform above or below expectations [20]. Combining ability analysis is often implemented through diallel, line × tester or North Carolina designs [21]. A complete diallel analysis was not feasible in guineagrass because apomixis prevents reciprocal crosses. North Carolina design methods enable breeders to dissect genetic variance into additive and non-additive components, thereby facilitating the identification of superior parents and predicting the performance of potential hybrids [22,23,24]. When GCA effects predominate, additive gene action is more important, and selection of parents or early segregating generations is effective. In opposition, a predominance of SCA suggests strong dominance or epistasis, favoring hybrid development strategies [23]. This analytical framework has been successfully applied across a wide range of crops to identify genotypes combining high yield, resilience, and quality attributes under contrasting environments [22,23]. Evidence from sorghum sudangrass, oat, maize, and alfalfa demonstrates that combining ability analysis is also highly effective for dissecting genetic control of forage yield and nutritional quality [25,26,27,28]. Studies in pearl millet have also demonstrated substantial differences in GCA and SCA among parents and hybrid combinations [29]. Additionally, multi-location analyses in maize and other cereals highlight the importance of exploring G × E and variance components using mixed models to improve the precision of GCA/SCA estimation [30].

In tropical forage species such as *Urochloa*, combining ability analyses have proven valuable for selecting parental lines with high general combining ability for biomass yield and forage quality, and for identifying specific hybrid combinations that exhibit favorable heterosis and adaptability to environmental stress [15]. Comparable outcomes have been reported in sorghum hybrids and maize dual-purpose systems, where combining ability parameters have guided parental selection under drought and other abiotic stresses [26,31]. In the context of *M. maximus*, the application of combining ability analysis represents a powerful approach to exploit the limited sexual gene pool, optimize the use of apomictic and sexual parents, and guide the selection of testers and superior hybrids with enhanced yield potential and forage quality.

Although substantial progress has been achieved in characterizing guineagrass germplasm, information on the combining ability remains scarce. Evaluating general and specific combining abilities for agronomic and nutritional quality traits is crucial for determining parental performance, identifying superior testers for hybridization, and designing an appropriate breeding scheme. This study tested two hypotheses: first, that agronomic and forage quality traits in the evaluated guineagrass NCII population are controlled by both average parental and specific family effects; and second, that apomictic male parents differ in combining ability, allowing the identification of promising tester candidates. Therefore, this study aimed to characterize the genetic architecture of agronomic and nutritional quality traits in guineagrass using a NCII mating design. Specifically, male-parent GCA, female-parent average effects, SCA, variance components, heritability, and genetic interaction with locations were estimated to identify promising apomictic testers and superior F_1_ families within the evaluated population, environments, and traits.

## 2. Results

### 2.1. Variance Components and Genetic Architecture

All results are presented in MS1 and MS2 for agronomic and nutritional quality traits, respectively. The NCII was incomplete because not all expected female × male combinations produced evaluable F_1_ families. From the expected 243, 175 families (Table 1) were retained for the analyses after filtering for known parentage, apomictic status, and phenotypic records. Therefore, estimates should be interpreted within the structure of an incomplete factorial NCII design. The incomplete NCII structure was handled using mixed models, which are appropriate for unbalanced data and allow estimation from all observed families. Because the precision of the predictions of the effect of parentals depends on family representation, tester rankings were interpreted in the context of the number and distribution of available crosses. The NCII mixed model showed that both average parental effects and specific family effects contributed to the performance of the F_1_ families. Variance components were calculated to estimate the relative importance of additive and non-additive types of gene action. The variance components estimated by the model are presented in Table 2, while Table 3 presents the causal additive variance and the variance associated with dominance or SCA, calculated according to the NCII framework [24]. This distinction is important because the components estimated directly by the model describe male parent GCA, female parent average effect and SCA, while the NCII variances are derived genetic parameters obtained by rescaling those model components [32].

For agronomic traits, GCA explained the largest proportion of the main genetic variance. The sum of GCA for male and female parent average effects accounted for 61.0% of the genetic variance in visual biomass score, 60.3% in drone biomass, 58.6% in plant cover, and 70.6% in plant height (Table 2). However, SCA also explained an important fraction of the main genetic variance. For agronomic traits, SCA ranged from 29.4% for plant height to 41.4% for plant cover (Table 2). Therefore, the results do not support a purely additive genetic control. Instead, hybrid performance was explained by both average parental effects and specific mother × father effects. Similar results were obtained in other forages and crop species, where both GCA and SCA contributed to biomass, yield or quality traits [27,28,29,33].

Related to nutritional quality traits, the predominance of GCA was stronger. Neutral detergent fiber showed the highest GCA proportion, with 80.1% of the main genetic variance explained by the effects of male and female parents. Crude protein also showed a high GCA contribution, with 73.5%. Finally, acid detergent fiber was also mainly explained by GCA, with 63.7%, but it showed the largest SCA contribution among the quality traits, with 36.3% of the genetic variance explained by SCA (Table 2). Therefore, selection based on average parental effects should be useful for improving nutritional quality.

The causal variances derived from NCII provided complementary information on the genetic architecture (Table 3). Causal additive variance was approximated from the male GCA and female parent average effect components, whereas variance from dominance/SCA was derived from the male × female interaction. For plant height, crude protein, and neutral detergent fiber, the additive variance derived from NCII was larger than the dominance/SCA variance. For the visual biomass score, plant cover, drone biomass, and acid detergent fiber showed a relatively higher dominance/SCA component (Table 3).

A relevant interpretation emerged when the variance components were read at two levels. In the mixed model, GCA explained the largest proportion of the main genetic variance for most agronomic and nutritional quality traits (Table 2). However, the NCII causal components showed that additive variance and dominance variance were similar in magnitude for several traits (Table 3).

Related to the genetic effects × location component (G × L in Table 2), this was estimated only for agronomic traits. The total genetic × location proportion ranged from 10.2% for visual biomass score to 21.7% for drone biomass (Table 2). This indicates that genetic effects varied moderately among locations, although most of the genetic signal remained associated with the main genetic components [27,31].

### 2.2. Heritability Estimates and Reliability of Selection

The heritability estimates showed that selection reliability depended strongly on the level at which selection was applied. Individual narrow-sense heritability from the NCII framework, *h*^2^*_i_*, was lower than family-mean heritability for all traits (Table 4). For agronomic traits, *h*^2^*_i_* ranged from 0.26 to 0.32, while for nutritional quality traits, evaluated only in Palmira, it ranged from 0.12 to 0.20.

In contrast, full-sib family-mean broad-sense heritability, *H*^2^*_FS_*, was higher because family means reduced the influence of residual and micro-environmental variation. For agronomic traits, *H*^2^*_FS_* was high, ranging from 0.89 for drone biomass to 0.95 for visual biomass score. Plant height and plant cover also showed high values, close to 0.91 (Table 4).

For nutritional quality traits, which were evaluated only in Palmira, *H*^2^*_FS_* was moderate to high, but lower than for agronomic traits. Neutral detergent fiber showed the highest value, followed by acid detergent fiber and crude protein (Table 4). Although the selection for nutritional quality is feasible, the precision was lower than for productivity traits. The observed difference is expected, in part, because nutritional quality was evaluated at a single location, whereas agronomic traits were evaluated across locations.

Male half-sib family-mean narrow-sense heritability, *h*^2^*_HSM_*, was high for both agronomic and nutritional quality traits (Table 4). The relevance of these results lies in the fact that one of the main objectives of the study was to identify promising apomictic testers. High *H*^2^*_FS_* indicates that male GCA was estimated with good reliability. Therefore, selection of apomictic male parents based on GCA is well supported by the data.

Female half-sib family-mean narrow-sense heritability, *h*^2^*_HSF_*, was more variable. For agronomic traits, *h*^2^*_HSF_* was lower than *h*^2^*_HSM_*, suggesting that selection among sexual female parents is possible but should be based on family-level information. For nutritional quality traits, *h*^2^*_HSF_* was relatively high for neutral detergent fiber and acid detergent fiber, but lower for crude protein (Table 4). This suggests that female-parent effects may be useful for improving fiber quality within the evaluated population, although they should not be interpreted as pure nuclear GCA because reciprocal crosses were not available.

### 2.3. General Combining Ability and Tester Selection

**Figure 1:**
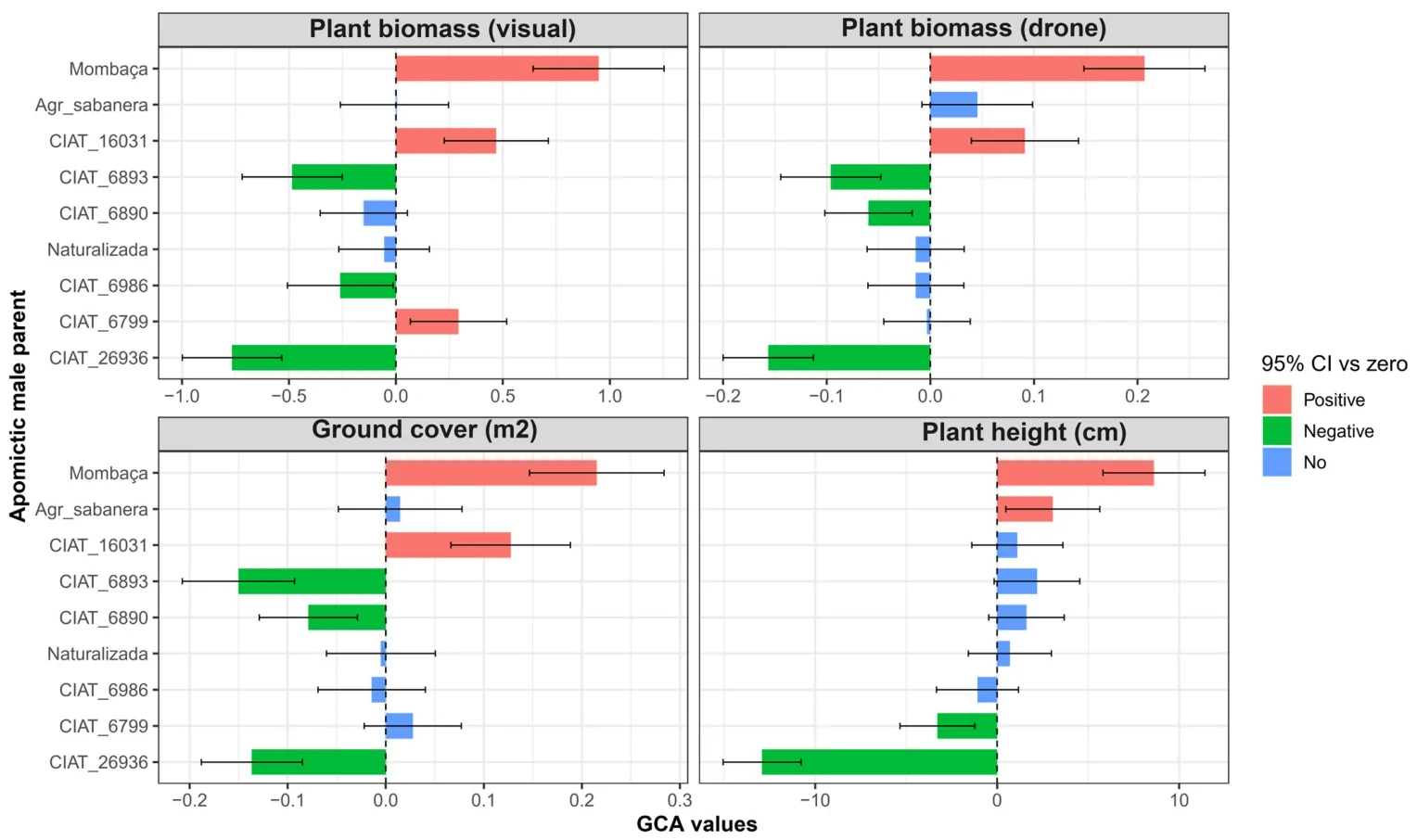
General combining ability of apomictic male parents for agronomic traits. Bars represent GCA estimates and error bars represent approximate 95% confidence intervals. Colors indicate whether the interval is positive, negative, or overlaps zero.

**Figure 2:**
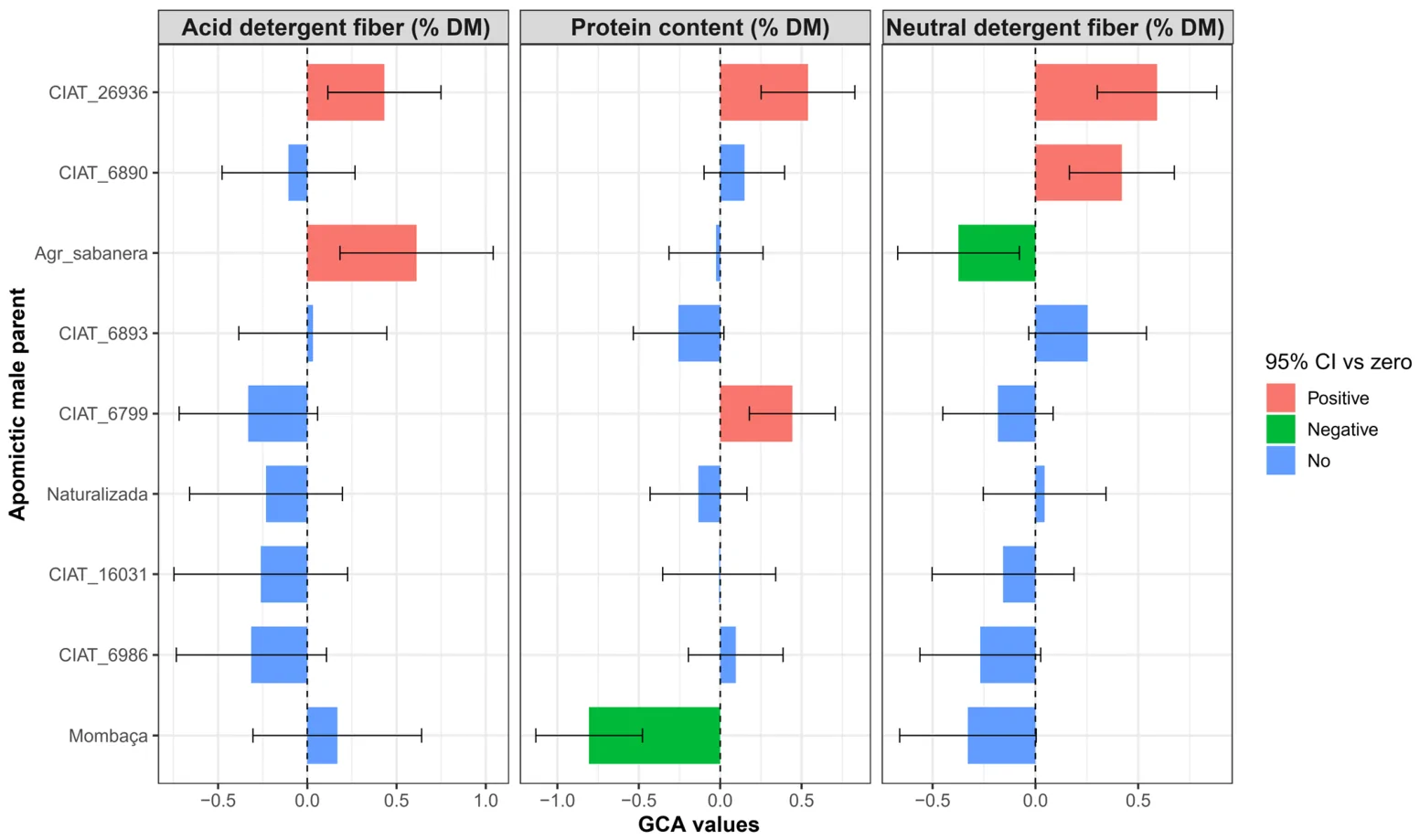
General combining ability of apomictic male parents for nutritional quality traits. Bars represent GCA estimates and error bars represent approximate 95% confidence intervals. Colors indicate whether the interval is positive, negative, or overlaps zero.

The GCA predictions showed clear differences among the apomictic male parents (Figure 1 and Figure 2; MS1 and MS2). For agronomic traits, the most favorable predicted male-parent GCA values were observed for Mombaça. It occupied the first position in ranking for visual biomass score, drone biomass, plant cover and plant height. Consequently, this pattern demonstrates its capacity to transfer favorable average effects for agronomic traits to its progeny.

This result supports the use of Mombaça as the main apomictic tester for recurrent selection focused on forage productivity. A tester with high and stable GCA is useful because it helps identify sexual female parents with favorable average performance. This principle is consistent with recurrent selection schemes in tropical forage grasses, where sexual populations are crossed with apomictic testers, and selected sexual parents are recycled into the next cycle [15].

CIAT_16031 also showed favorable GCA for visual biomass score, drone biomass, and plant cover (Figure 1). Therefore, it can be considered a complementary productivity tester, especially for biomass and plant cover. However, its GCA for plant height was less favorable, suggesting that its main contribution is related more to biomass and cover than to plant height. Agr_sabanera showed a more specific profile, with favorable GCA for plant height and drone biomass. In contrast, CIAT_26936 showed low or negative GCA for most productivity traits, suggesting limited value as a general productivity tester.

For nutritional quality traits (Figure 2), the favorable direction depended on the trait. For neutral and acid detergent fiber, lower values are desirable, so negative GCA values are favorable. For crude protein, higher values are desirable, so positive GCA values are favorable. Under this criterion, Agr_sabanera, Mombaça, CIAT_6986, CIAT_6799 and CIAT_16031 showed favorable GCA for reducing neutral detergent fiber. For acid detergent fiber, the most favorable male parents were CIAT_6799, CIAT_6986, CIAT_16031 and Naturalizada. For crude protein, the most favorable male parents were CIAT_26936 and CIAT_6799, followed by CIAT_6890 and CIAT_6986 (Figure 2; MS2).

Not a single apomictic parent was superior for all breeding objectives. Mombaça showed the most favorable predicted male parent GCA profile for productivity and also contributed to lower neutral detergent fiber. However, it did not show the most favorable predicted effects for reducing acid detergent fiber or increasing crude protein. For forage nutritive quality traits, CIAT_6799 showed favorable predicted effects, particularly for reducing acid detergent fiber and increasing crude protein, whereas CIAT_6986 showed a balanced profile across fiber and protein traits.

Female-parent average-effect predictions also showed useful variation (MS1 and MS2). Some sexual female parents showed favorable average effects across several traits and should be considered for recurrent improvement of the sexual population. However, because reciprocal crosses were not available, the female-parent effect should not be interpreted as pure nuclear GCA. It should be treated as a female-parent average effect that may include several sources associated with the female parent.

### 2.4. Specific Combining Ability and Superior F_1_ Families

**Figure 3:**
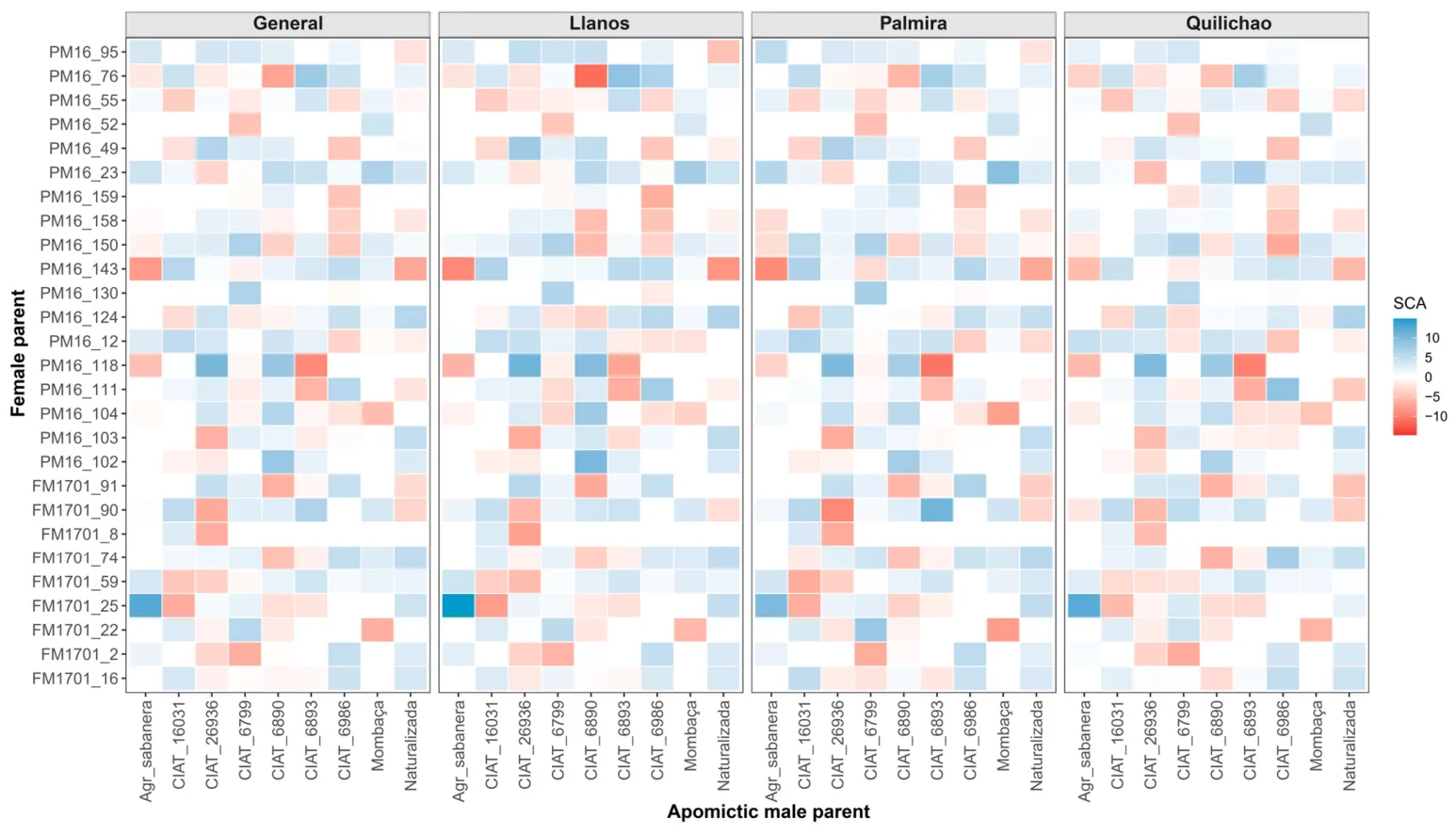
Specific combining ability of F_1_ families for plant height in each location and across locations (general). Blue indicates positive SCA, red indicates negative SCA, and white indicates values close to zero.

**Figure 4:**
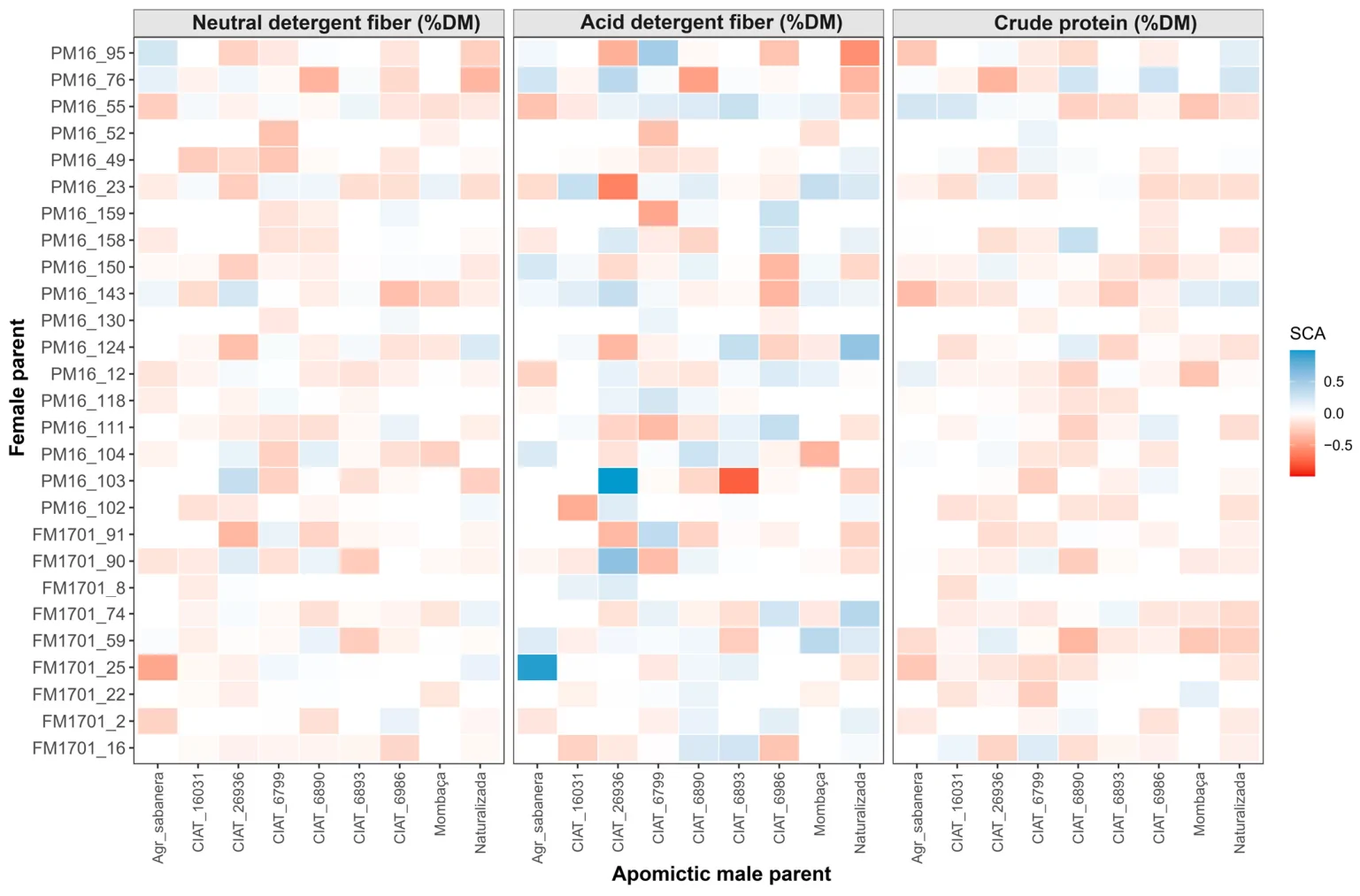
Specific combining ability of F_1_ families for nutritional quality traits evaluated in the Palmira location. Blue indicates positive SCA, red indicates negative SCA, and white indicates values close to zero.

SCA predictions were used to identify families that performed above or below expectation based on male-parent GCA and female-parent average effects (Figure 3 and Figure 4; MS1 and MS2). This interpretation follows the classical definition of SCA as the deviation of a specific cross from the average effects of its parents [17,19,20]. For agronomic traits, some families combined favorable parental GCA with positive SCA, including PM16_23 × Mombaça, PM16_52 × Mombaça and PM16_150 × Mombaça. Other families were outstanding mainly because of SCA, such as FM1701_25 × Agr_sabanera, which showed high SCA for several productivity traits. These results indicate that specific parental combinations contributed to F_1_ family performance beyond the average effects of the corresponding parents.

For nutritional quality traits, SCA also helped to identify favorable specific families (Figure 4, MS2). For neutral detergent fiber, PM16_49 showed favorable combinations with CIAT_6799, Agr_sabanera, CIAT_16031, Mombaça and CIAT_6986. For acid detergent fiber, favorable families included PM16_95 × Naturalizada, PM16_95 × CIAT_6986, PM16_52 × CIAT_6799, PM16_150 × CIAT_6986 and PM16_111 × CIAT_6799. For CP, promising families included PM16_76 × CIAT_6890, FM1701_16 × CIAT_6799, PM16_76 × CIAT_6986, PM16_55 × CIAT_26936 and PM16_49 × CIAT_6799. These results suggest that GCA is useful for choosing parents, while SCA is useful for identifying specific F_1_ families that deserve validation.

This distinction is especially important in apomictic breeding. In sexual crops, high SCA is mainly useful to identify hybrid combinations that must be recreated by crossing. In apomictic forages, favorable family-specific effects may have additional value because superior genotypes can potentially be fixed through apomixis [6,10,15].

## 3. Discussion

### 3.1. Genetic Architecture and Combining Ability

The results of this study should be interpreted within the specific context of the NCII population, the environments, and the traits evaluated. The study provides quantitative evidence to guide future strategies in the breeding of guineagrass, *M. maximus*. The main finding was that agronomic and nutritional quality traits showed a mixed genetic architecture. Male parent GCA and female parent average effects explained the largest proportion of the main genetic variance for most traits, ranging from 58.6% to 80.1%. In this context, these parental components reflect the average contribution of each parent across crosses, while SCA captures the extent to which specific male × female contributions deviated from their expected performance based on parental average effects [17,19,20,34]. However, SCA also explained an important proportion of the genetic variance. Thus, the results do not support a purely additive interpretation of these traits, but rather a genetic architecture in which both average parental effects and specific family effects contributed to F_1_ performance.

This interpretation becomes clearer when the GCA/SCA proportions are considered together with the NCII-derived causal variance components. This result is not contradictory. The GCA and SCA percentages describe the relative importance of parental average effects and specific family effects in the fitted model. In contrast, the NCII components express these same sources of variation after rescaling them according to the expected covariance structure of the NCII design [20,24,32]. For this reason, the relatively high GCA proportions and the similar magnitude of additive and dominance/SCA-related variance observed for some traits are not contradictory. They indicate that parental average effects were important, but that specific male × female combinations also contributed to hybrid performance.

These results differ in part from those reported in other grasses; for example, in *Urochloa* spp., non-additive effects were predominant for agronomic and nutritional quality traits and SCA was significant for all traits [35]. These differences may be related to the genetic constitution of the parental populations, the reproductive biology of the species, the mating design, and the set of traits analyzed. In the population analyzed, GCA was important for most traits, but SCA remained relevant, especially for plant cover, drone biomass, plant biomass (visual), and acid detergent fiber. This suggests that breeding decisions should not rely only on parental average effects, but should also consider the performance of specific F_1_ families.

The heritability estimates also supported this interpretation. Full-sib family-mean narrow-sense heritability, *h*^2^*_FS_*, was moderate for all traits (Table 4). This estimator includes only GCA components and therefore reflects the reliability of family selection based on average parental effects. The difference between *h*^2^*_FS_* and *H*^2^*_FS_* shows that SCA also contributed to family performance. Thus, GCA is useful for selecting testers and improving the sexual recurrent population, while *H*^2^*_FS_* supports the identification of superior F_1_ families based on their total family value.

Among the heritability estimators, *h*^2^*_HSM_*, *h*^2^*_HSF_*, *h*^2^*_FS_*, and *H*^2^*_FS_*. The high *h*^2^*_HSM_* supports the selection of apomictic testers based on GCA. The moderate to high *h*^2^*_HSF_* supports the selection and recombination of superior sexual female parents. The moderate *h*^2^*_FS_* indicates that average parental effects are useful at the family level, while the high or moderate *H*^2^*_FS_* indicates that adjusted F_1_ family means are reliable for validation. This is particularly important in apomictic breeding, where superior family-specific effects may have direct value if they can be fixed through apomictic reproduction [10,15,36].

### 3.2. Tester Selection and Breeding Implications

Among the male parents evaluated, Mombaça, CIAT_6962, was the most consistent tester for productivity traits. It showed the highest GCA for visual biomass score, drone biomass, plant cover and plant height, and it also showed high stability across locations. Therefore, Mombaça should be considered a promising tester for productivity traits rather than a universal tester for all breeding objectives.

The high male half-sib family mean heritability, *h*^2^*_HSM_*, indicates that the GCA of apomictic male parents was estimated with high reliability. This supports the use of apomictic testers to evaluate and improve the sexual population. This strategy is consistent with recurrent selection schemes used in apomictic tropical forage grasses, especially in interspecific *Urochloa*, where sexual populations are crossed with an apomictic tester, superior sexual parents are identified through progeny performance and selected sexual parents are recombined for the next cycle [14,15]. In the present study, the moderate to high female half-sib family mean heritability, *h*^2^*_HSF_*, indicates that selection among sexual female parents is feasible. However, this selection should be based on family-level information rather than individual observations.

Also, the results showed that no single apomictic parent was superior for all breeding traits. This is important because productivity and nutritional quality did not identify the same best male parents. Although Mombaça showed favorable predicted GCA for productivity and contributed to lower neutral detergent fiber, it did not show the most favorable predicted effects for reducing acid detergent fiber or increasing crude protein. In contrast, CIAT_6799 and CIAT_6986 showed a more balanced profile across fiber and protein content. Thus, Mombaça appears more suitable for productivity traits, while CIAT_6799 and CIAT_6986 should be considered preliminary donors to improve quality traits under Palmira conditions. This agrees with the idea that a single universal tester is not always optimal when several breeding objectives are considered [35].

The results for nutritional quality traits should therefore be interpreted as specific to the Palmira environment. Therefore, no genotype × location assessment was possible for these traits. The observed quality profiles provide useful preliminary evidence for parental selection, but they should not be interpreted as broad multi-environment recommendations. Additional trials across contrasting environments will be needed to determine whether CIAT_6799 and CIAT_6986 maintain favorable nutritive quality effects under different edaphoclimatic conditions.

The results also show that recurrent selection based on GCA should be complemented with family validation based on SCA. This indicates that specific parental complementation can generate superior F_1_ performance even when the parents do not show the highest average effects, as also reported in line × tester studies where non-additive effects contribute to hybrid performance [22,23,28]. Some families performed better than expected from the average effects of their parents. This was observed for both agronomic and nutritional quality traits. These families are important because they may contain favorable specific combinations that would not be detected by GCA alone. This agrees with line × tester studies in which non-additive effects contribute to hybrid performance [25,26]. In practical terms, male parent GCA should guide tester selection, female parent average effects should support recurrent improvement of the sexual population, and SCA should guide the identification of superior families.

This architecture has direct implications for breeding. Recurrent selection based on SCA can be used to increase the frequency of favorable alleles in the sexual recurrent population [37]. At the same time, F_1_ families with favorable SCA can be validated as candidate apomictic hybrids. This is especially relevant in apomictic forages. In sexual crops, favorable SCA is useful to identify hybrid combinations, but those combinations usually need to be replicated by controlled crossing. In apomictic forages, superior family effects may have additional value because elite genotypes can potentially be fixed through apomictic seed reproduction [38]. Thus, guineagrass offers the possibility of accumulating additive effects through recurrent selection and exploiting favorable non-additive or family-specific effects through apomictic fixation [6,15,38].

### 3.3. Limitations and Future Directions

Several limitations should be considered when applying these results. First, the NCII design was incomplete because not all expected female × male combinations produced evaluable F_1_ families. The incomplete factorial structure was handled using mixed models, which are appropriate for unbalanced data because they use all available family information. However, the precision of parental-effect predictions depends on the number and distribution of observed crosses. Therefore, variance components, GCA predictions, SCA predictions, and tester rankings should be interpreted in the context of the observed family representation in the incomplete NCII design.

Second, female-parent average effects should not be interpreted as pure nuclear GCA because reciprocal crosses were not available due to the reproductive constraints of the species. These effects may include nuclear combining ability, maternal effects, cytoplasmic effects, seed-related effects, or other female-parent-associated sources of variation.

Third, nutritional quality traits were evaluated in one location only, whereas agronomic traits were evaluated across locations. Therefore, the interpretation of results for nutritional quality traits applies only to the Palmira environment. These results should be considered a first indication of parental and family value for nutritive quality, and additional multi-environment evaluations will be required to confirm the stability of crude protein, neutral detergent fiber, and acid detergent fiber.

Despite these limitations, the results provide a useful quantitative framework for guiding the next stages of guineagrass breeding. A practical breeding scheme can be organized in two phases. In the short term, the program can apply recurrent selection assisted by testers. The complete synthetic sexual population should be crossed with the best testers identified in this study. For productivity traits, Mombaça should be considered as the main tester candidate. For nutritional quality, CIAT_6799 and CIAT_6986 should be included as preliminary tester candidates, but their performance must be validated across additional environments before broad recommendation.

Sexual female parents should then be selected using progeny performance, SCA, and stability across environments using the desired gains index selection, which allows the selection of genotypes when there are multiple traits and more when these traits present a marked trade-off, such as production and nutritional quality [39]. Selected sexual parents can be recombined to form the next cycle of the sexual population. In parallel, superior F_1_ families with high performance and favorable SCA should be validated as potential apomictic cultivar candidates.

In future cycles, optimal contribution selection (OCS) could be implemented as a complementary strategy once enough phenotypic and genomic information becomes available [40]. OCS should not be viewed as a replacement for recurrent selection assisted by testers, but as a tool to balance genetic gain and the preservation of genetic diversity. This is particularly relevant for guineagrass because the genetic base of cultivated materials is narrow and synthetic sexual populations are still under development [16]. Simple truncation selection could rapidly increase the use of a few superior parents, such as Mombaça for productivity, but this could also reduce diversity and increase coancestry. OCS provides a way to balance genetic gain and diversity by deciding not only which parents are selected, but also how much each parent contributes to the next cycle [40,41,42]. This would be useful to maintain long-term genetic variability while still exploiting the best GCA sources identified in this study [40,42].

The NCII population evaluated here was an experimental subset and not the complete tetraploid synthetic sexual population to be improved. Therefore, before implementing OCS, the complete sexual population should be evaluated through crosses with the selected testers, and SNP marker information should be collected for both parents and derived apomictic hybrids. This genomic information would allow the construction of a genomic relationship matrix and the estimation of optimal parental contributions while controlling relatedness and preserving genetic diversity [42].

A complementary analysis of heterosis and genetic distance will also be important. SCA indicates whether a family performs above or below expectation based on parental average effects, but it does not directly quantify heterosis. A formal heterosis analysis would require comparing adjusted F_1_ performance with adjusted mid-parent and better-parent values. Heterosis has been reported in tropical forage grasses for biomass and nutritional quality traits, but systematic analyses in *M. maximus* using synthetic sexual populations are still limited [8]. Estimating heterosis in the full set of F_1_ families would help identify specific crosses with true hybrid vigor and clarify the relationship between parental divergence, SCA and hybrid performance. This will be discussed in the next paper.

Once SNP data are available, genetic distances among the sexual and apomictic parents can be estimated and used to guide future crossing decisions. In the present breeding context, genomic distances could help determine whether genetically divergent crosses tend to show higher SCA or heterosis. They could also support OCS by providing a genomic relationship matrix to control coancestry while maintaining diversity in the recurrent population [40].

## 4. Materials and Methods

### 4.1. Plant Material, Mating Design and Identification of Apomictic Progeny

A North Carolina II factorial mating design was implemented to evaluate additive genetic effects and to assess the contribution of male parents across environments. A subset of 27 tetraploid sexual genotypes was selected from a population synthesized by crossing a sexual genotype population (USDA GenBank PI 570664) with 93 apomictic genotypes from CIAT GenBank. These sexual genotypes were used as female parents (♀), and nine apomictic genotypes were selected as candidate male testers (♂) based on prior agronomic evaluations ([43], Table 5).

Guineagrass is a wind-pollinated species; for this reason, nine spatially isolated pollination blocks separated by >200 m were arranged, each assigned to a single male parent. Each block consisted of clonal plants of the apomictic male parent distributed evenly across the plot, while clones of the 24 sexual female parents were interspersed uniformly within the block. This configuration surrounded each sexual female with a corresponding apomictic tester, maximizing open cross-pollination while minimizing pollen contamination. After flowering, seeds were hand-harvested individually from each female parent within each block. The expected factorial structure included 243 F_1_ families (27 ♀ × 9 ♂); however, only 175 F_1_ families were obtained after filtering for seed availability, apomictic status and survivorship (Table 1).

Seeds from each family were planted in sand, maintaining family identity. After 45 days, seedlings were transplanted into containers filled with a soil:sand (3:1) substrate. The resulting F_1_ population was maintained in a greenhouse. To confirm the reproductive behavior of the hybrids, the molecular marker p779/p780 was used to detect the ASGR-BBML allele associated with apomixis in Panicoideae (Poaceae) [36]. Identifying apomictic genotypes ensured the validation of true hybrids in subsequent trials, since some individuals classified as sexual could originate from self-pollination or crosses between two sexual genotypes.

### 4.2. Multi-Environmental Trial

Apomictic genotypes were transplanted into pots and maintained under greenhouse conditions. Once sufficient tillers developed, clonal propagation was carried out in 3-inch containers, maintaining genotype identity. After 45 days, clones were transplanted to field sites. Hybrids and their parental lines were evaluated in three contrasting environments in Colombia: Llanos (4°17′55.3″ N, 72°25′06.2″ W), Quilichao (3°05′15.1″ N, 76°27′18.3″ W), and Palmira (3°30′20.6″ N, 76°21′17.3″ W). These sites were selected for their contrasting edaphoclimatic conditions. Llanos (Meta Department): high rainfall, unimodal regime with a dry season from December to March, high solar radiation, acidic soils with low fertility and high aluminum saturation. Quilichao (Cauca Department): Andean influence, higher cloudiness, medium temperature, volcanic soils rich in organic matter, low drainage, bimodal rainfall. Palmira (Valle del Cauca Department): bimodal rainfall, located on the Cauca River alluvial plain, clay soils, high natural fertility due to volcanic ash deposits.

Each location followed Federer’s augmented block design [44]. Twenty-six blocks were established per site, each comprising 81 experimental units, for a total of 2106 plots per location. Each block included 79 unique genotypes and 3 recurrent checks, systematically distributed to estimate experimental error and adjust block effects. Within each block, the 79 genotypes were randomly selected from a candidate population including F_1_ hybrids, 24 sexual parents, and 9 apomictic parents. The checks corresponded to widely adopted commercial cultivars: Mombaça (CIAT 6962), Tanzania 1 (CIAT 16031), and Tanzania 2 (CIAT 16031). Plant spacing was 2 m between rows and 2 m between plants to minimize competition among units. To minimize border effects, a border row of cv. Mombaça was established around each trial.

### 4.3. Evaluated Traits

Five evaluations were conducted per site: one initial evaluation 65 days after planting and four regrowth evaluations conducted every 35 days. Regrowth assessments were conducted according to rainfall regimes, two during the wet season and two during the dry season. Traits were evaluated following the CropOntology standards [45]. Visual plant biomass was scored on a 1–9 scale based on aboveground biomass accumulation (1 = lowest, 9 = highest). Plant height (cm) was measured in centimeters from the soil surface to the tip of the foliage, excluding inflorescences. Plant cover (m^2^) was quantified via drone imagery (DJI Phantom 4 RTK, SZ DJI Technology Co., Ltd., Shenzhen, China) processed in Agisoft Metashape Professional version 2.3.1 (Agisoft LLC, St. Petersburg, Russia). Drone biomass (cm^3^) was estimated by multiplying plant cover by plant height, providing an integrated proxy of canopy size and vertical growth.

Nutritional value was assessed using crude protein, neutral detergent fiber, and acid detergent fiber concentrations, expressed as percentage of dry matter (% DM). These traits were measured exclusively in samples collected in Palmira during a single evaluation period. Chemical analyses were performed at the CIAT Forage Quality and Animal Nutrition Laboratory. Acid and neutral detergent fibers were analyzed using an ANKOM 2000 Fiber Analyzer (ANKOM Technology, Macedon, NY, USA) [46]. Crude protein was quantified using a Kjeltec 8100 distillation unit (FOSS Analytical A/S, Hillerød, Denmark) according to AOAC 2001.11 [47]. All measurements were conducted in duplicate, and mean values were used for subsequent statistical analyses.

### 4.4. Statistical Analysis

To evaluate the combining ability of the apomictic tester candidates used as male parents, a mixed factorial NCII model was fitted using only F_1_ hybrid families with known female and male parents. Before model fitting, parental entries were removed, and check varieties were excluded. The analysis was conducted using ASReml-R version 4.6.1 [48]. Location was considered as the main environment, whereas regrowth date was fitted as an effect nested within location. The statistical model used was: *Y_ijkpq_* = *µ* + *L_i_* + *R_j__(i)_* + *M_p_* + *F_q_* + (*MF*)*_pq_* + (*LM*)*_ip_* + (*LF*)*_iq_* + (*LMF*)*_ipq_* + *Ɛ_i_*. Where *Y_ijkpq_* is the observation of the family derived from male parent *p* and female parent *q*, evaluated in location *i*, at regrowth or evaluation *j* within that location. *μ* is the overall mean; *L_i_* is the fixed effect of location (environment); *R_j_*_(*i*)_ is the fixed effect of regrowth or evaluation nested in the location; *M_p_* is the random effect of the male parent, interpreted as male parent GCA; *F_q_* is the random effect of the female parent, interpreted as the female-parent average effect. Because reciprocal crosses were not available, this term was not interpreted as purely nuclear female GCA; (*MF*)*_pq_* is the male × female interaction, interpreted as specific combining ability; (*LM*)*_ip_* and (*LF*)*_iq_* are the interactions of male parent GCA × location and female parent average effect × location; (*LMF*)*_ipq_* is the SCA × location interaction; and *ε_ijkpq_* is the residual error [49].

Male parent, female parent, SCA, and their interactions with location were fitted as random effects, assuming independent normal distributions with mean zero and trait-specific variances. Residual variance was allowed to differ among locations to account for possible differences in experimental precision across environments. For nutritional quality traits, the analysis was conducted in a single location. Therefore, location and its interactions were removed from the model. Residual variance was allowed to differ among batches. Male parent GCA was estimated from the random effect *M_p_*, whereas the female-parent average effect was estimated from *F_q_*. SCA was estimated from the *M_p_ × F_q_* interaction, allowing the identification of families that performed above or below expectation based on the average contribution of their parents. Because this was a one-way mating design, the female-parent effect was interpreted with caution, as it may include not only nuclear combining ability but also effects associated with the female parent.

To assess whether combining ability varied across locations, the variance components of the *L* × *M*, *L* × *F*, and *L* × *M* × *F* interactions were examined. The *L* × *M* component was used to determine whether the GCA of apomictic male parents was stable or dependent on location. In contrast, *L* × *M* × *F* was used to evaluate how SCA changes across locations. As complementary evidence, likelihood ratio tests were performed by comparing the full model with reduced models in which the father GCA × location, mother GCA × location, and SCA × location terms were removed individually. These comparisons were used to evaluate whether the inclusion of each interaction improved model fit. In addition, a global SCA test was performed by comparing the full model with a reduced model excluding the male × female interaction and its interaction with location. Because nutritional quality traits were measured only in Palmira, location and genotype × location interaction terms were not estimable for crude protein, neutral and acid detergent fiber. Consequently, inference for these traits is restricted to the evaluated location.

The relative importance of additive and non-additive effects was assessed using the proportion of variance explained by GCA and SCA. The proportion associated with GCA was computed as: GCA(%) = 100 × [(*σ*^2^*_M_* + *σ*^2^*_F_*)/(*σ*^2^*_M_* + *σ*^2^*_F_* + *σ*^2^*_MF_*)], while the proportion of SCA was estimated as SCA(%) = 100 × [*σ*^2^*_MF_*/(*σ*^2^*_M_* + *σ*^2^*_F_* + *σ*^2^*_MF_*)], where *σ*^2^*_M_* is the male parent GCA variance, *σ*^2^*_f_* is the female parent average effect or average female-parent variance, and *σ*^2^*_MF_* is the SCA variance. Baker’s ratio was also calculated as an overall measure of the relative predominance of GCA over SCA. In addition, additive causal variance was approximated as *σ*^2^*_A_* = 2(*σ*^2^*_M_* + *σ*^2^*_F_*), and variance related to dominance was approximated as *σ*^2^*_D_* = 4*σ*^2^*_MF_*. For agronomic traits evaluated across locations, additive × location and dominance/SCA × location variances were approximated as *σ*^2^*_A×L_* = 2(*σ*^2^*_LM_* + σ^2^*_LF_*) and *σ*^2^*_D_*_×*L*_ = 4*σ*^2^*_LMF_*, respectively [24].

Several heritability estimators were calculated to represent different selection levels (MS3). Individual narrow-sense heritability was calculated using the additive variance derived from mixed-model analysis. Individual broad-sense heritability was calculated using the sum of additive and dominance variances. In addition, female-parent and male-parent half-sib family-mean heritabilities were calculated to evaluate the reliability of selecting female parents and apomictic male testers based on their average effects. A pooled half-sib heritability was also computed as a general measure of parental selection reliability. Finally, full-sib family-mean heritability was calculated in the narrow and broad sense. The narrow-sense full-sib family-mean heritability included only GCA components, whereas the broad-sense full-sib family-mean heritability included GCA and SCA components. For agronomic traits, broad-sense full-sib family-mean heritability was calculated using the variance components from the mixed model: *H*^2^*_FS_* = (*σ*^2^*_M_* + *σ*^2^*_F_* + *σ*^2^*_MF_*)*/*(*σ*^2^*_M_* + *σ*^2^*_F_* + *σ*^2^*_MF_* + (*σ*^2^*_LM_* + *σ*^2^*_LF_* + *σ*^2^*_LMF_*)/*nl* + *σ*^2^*_e_/nlr*), where *σ*^2^*_LM_*, *σ*^2^*_LF_*, *σ*^2^*_LMF_*, are the father GCA × location, mother GCA × location, and SCA × location variances, respectively; *n_l_* is the number of locations; *r* is the average number of observations per family within location; and *σ*^2^*_e_* is the average residual variance. For nutritional quality traits, which were evaluated in a single location, the location interaction terms were omitted from the denominator.

## 5. Conclusions

This study showed that agronomic and nutritional quality traits in the evaluated guineagrass (*Megathyrsus maximus*) NCII population were controlled by both average parental effects and specific family effects. Male-parent GCA explained a large proportion of the main genetic variance, supporting the use of apomictic testers for recurrent selection of the sexual population. However, SCA also contributed to family performance, indicating that adjusted family means and specific cross performance should be considered when identifying superior F_1_ families.

Within the evaluated population and environments, Mombaça (CIAT_6962) showed the most favorable predicted male-parent GCA profile for productivity-related traits. In contrast, CIAT_6799 and CIAT_6986 showed preliminary favorable profiles for forage nutritive quality traits under Palmira conditions.

The results support a breeding strategy based on recurrent selection assisted by testers, family validation, and subsequent multi-environment analysis. Future work should validate tester performance across additional environments and integrate heterosis analysis, genetic-distance estimates, and genomic information to support long-term genetic gain while preserving diversity in guineagrass breeding populations.

## Figures and Tables

**Table 1:** Crossing success and number of evaluable F_1_ families in the incomplete North Carolina II mating design.

Parental	Agr_sabanera	CIAT_16031	CIAT_26936	CIAT_6799	CIAT_6890	CIAT_6893	CIAT_6986	Mombaça	Naturalizada	Total
**FM1701_16**		11	6	13	26	12	13		2	83
**FM1701_2**	14		1	1	16		11		1	44
**FM1701_22**		2	10	7	13			3		35
**FM1701_25**	45	9	2	10	4	12			13	95
**FM1701_59**	20	1	6	16	17	16	8	16	19	119
**FM1701_74**		6	9	8	21	16	7	20	10	97
**FM1701_8**		6	10							16
**FM1701_90**	13	2	15	20	17	6		2	7	82
**FM1701_91**			11	8	6	12	17		10	64
**PM16_102**		13	9		16	17			10	65
**PM16_103**			33	19	21	20	4		14	111
**PM16_104**	18		14	9	5	2	9	6		63
**PM16_111**		2	4	9	11	3	26		10	65
**PM16_118**	4		1	9	7	2				23
**PM16_12**	16	1	13	10	21	14	12	18	8	113
**PM16_124**		5	22	7	24	9	6	20	11	104
**PM16_130**				8			18			26
**PM16_143**	31	5	7	12	6	19	13	29	16	138
**PM16_150**	16	2	20	21	14	22	13	5	7	120
**PM16_158**	8		2	11	12		6		4	43
**PM16_159**				9	3		11			23
**PM16_23**	17	9	14	10	17	23	16	11	17	134
**PM16_49**		5	4	16	14		4		1	44
**PM16_52**				11				2		13
**PM16_55**	23	11	7	13	10	20	9	17	10	120
**PM16_76**	20	6	8	5	15	10	11		9	84
**PM16_95**	17		6	5	21		6		5	60
Total	262	96	234	267	337	235	220	149	184	2021

**Table 2:** Variance components and relative contribution of GCA, SCA and genetic interaction with location in the NCII analysis. *σ*^2^*_M_* = male GCA variance; *σ*^2^*_F_* = female parent average effect variance; *σ*^2^*_MF_* = male × female interaction variance, interpreted as SCA; *σ*^2^*_LM_* = male GCA × location variance; *σ*^2^*_LF_* = female parent effect × location variance; *σ*^2^*_LMF_* = SCA × location variance; *σ*^2^*_e_* = residual variance; GCA % = proportion of the main genetic variance explained by male and female parent effects; SCA % = proportion of the main genetic variance explained by SCA; Baker ratio = relative contribution of GCA to the main genetic variance; G × L % = proportion of the total genetic plus genetic × location variance explained by genetic interaction with location.

Trait	*σ* ^2^ *_M_*	*σ* ^2^ *_F_*	*σ* ^2^ *_MF_*	*σ* ^2^ *_LM_*	*σ* ^2^ *_LF_*	*σ* ^2^ *_LMF_*	*σ* ^2^ *_e_*	GCA %	SCA %	Baker Ratio	G × L % ^1^
Plant biomass (visual)	0.27	0.08	0.23	0.02	0.02	0.03	1.55	60.99	39.01	0.61	10.21
Plant height (cm)	33.56	6.29	16.61	4.43	4.12	5.09	231.44	70.58	29.42	0.71	19.46
Ground cover (m^2^)	0.01	0.00	0.01	0.00	0.00	0.00	0.08	58.57	41.43	0.59	16.75
Plant biomass (drone)	0.01	0.00	0.01	0.00	0.00	0.00	0.07	60.30	39.70	0.60	21.74
Neutral detergent fiber (%DM)	0.14	0.15	0.07	--	--	--	1.82	80.13	19.87	0.80	--
Acid detergent fiber (%DM)	0.15	0.22	0.21	--	--	--	3.79	63.72	36.28	0.64	--
Crude protein (%DM)	0.17	0.03	0.07	--	--	--	2.08	73.53	26.47	0.74	--

^1^ G × L (%) represents the proportion of the total genetic plus genetic interaction variance explained by genetic interaction with location. It includes father GCA × location, female-parent effect × location and SCA × location.

**Table 3:** NCII-derived additive and dominance variance components for agronomic and nutritional quality traits. *σ*^2^*_A_* NCII = causal additive genetic variance derived from the NCII framework; *σ*^2^*_D_* NCII = causal dominance variance derived from the NCII framework using the male × female interaction component; *σ*^2^_*A*×*L*_ = causal additive genetic variance associated with interaction with location; *σ*^2^_*D*×*L*_ = dominance or SCA-related variance associated with interaction with location.

Trait	*σ* ^2^ *_A_*	*σ* ^2^ *_D_*	*σ* ^2^_*A*×*L*_	*σ*^2^_*D*×*L*_ ^1^
Plant biomass (visual)	0.71	0.91	0.07	0.13
Plant height (cm)	79.70	66.44	17.11	20.34
Ground cover (m^2^)	0.03	0.05	0.01	0.01
Plant biomass (drone)	0.02	0.03	0.01	0.01
Neutral detergent fiber (%DM)	0.58	0.29	--	--
Acid detergent fiber (%DM)	0.74	0.85	--	--
Crude protein (%DM)	0.41	0.29	--	--

^1^ *σ*^2^*_A_* NCII was estimated from the male and female parent variance components. *σ*^2^*_D_* NCII was estimated from the male × female interaction component. For agronomic traits, *σ*^2^_*A*×*L*_ and *σ*^2^_*D*×*L*_ were estimated from the corresponding genetic × location interaction components. Nutritional quality traits were evaluated in a single location; therefore, genetic interaction with location was not estimated.

**Table 4:** Heritability estimates and stability ratios for agronomic and nutritional quality traits. *h*^2^*_i_* = individual narrow sense heritability from the NCII framework; *h*^2^*_HSF_* = female half sib family mean narrow sense heritability; *h*^2^*_HSM_* = male half sib family mean narrow sense heritability; *h*^2^*_HSP_* = pooled half sib family mean narrow sense heritability; *h*^2^*_FS_* = full sib family mean narrow sense heritability based on GCA components; *H*^2^*_FS_* = full sib family mean broad sense heritability based on GCA and SCA components; Male GCA stability = stability ratio of male GCA across locations; Female parent average effect stability = stability ratio of the female average effect across locations; SCA stability = stability ratio of SCA across locations.

Trait	*h* ^2^ *_i_*	*h* ^2^ *_HSF_*	*h* ^2^ *_HSM_*	*h* ^2^ *_HSP_*	*h* ^2^ *_FS_*	*H* ^2^ *_FS_*	Male GCA Stability	Female Average Effect Stability	SCA Stability ^1^
Plant biomass (visual)	0.32	0.49	0.92	0.76	0.58	0.95	0.95	0.81	0.88
Plant height (cm)	0.26	0.39	0.92	0.76	0.64	0.91	0.91	0.61	0.77
Ground cover (m^2^)	0.28	0.38	0.88	0.72	0.53	0.91	0.91	0.76	0.89
Plant biomass (drone)	0.26	0.28	0.88	0.71	0.54	0.89	0.89	0.54	0.81
Neutral detergent fiber (% DM)	0.2	0.71	0.86	0.78	0.53	0.66	--	--	--
Acid detergent fiber (% DM)	0.12	0.66	0.77	0.7	0.38	0.6	--	--	--
Crude protein (% DM)	0.12	0.3	0.91	0.7	0.41	0.56	--	--	--

^1^ Stability ratios were calculated only for agronomic traits evaluated across locations. Nutritional quality traits were evaluated in a single location; therefore, male GCA stability, female parent average effect stability and SCA stability were not estimated.

**Table 5:** Key characteristics of the apomictic parental lines used as tester candidates.

Genotype	Origin/Source	Morphotype	Key Traits/Description	Breeding Use
Agrosavia Sabanera	Agrosavia (Colombia)	Semi-erect	High yield, high protein content, shade tolerance.	Released cultivar
Tanzania (CIAT_16031)	Korogwe, Tanzania	Erect	Very leafy, moderate to high spittlebug resistance, moderate drought and cold tolerance.	Released cultivar
CIAT_26936		Semi-erect	High seed set potential.	Wild
CIAT_6799		Erect	Well adapted to acid soils with high Al saturation.	Wild
CIAT_6890	Kenia	Erect	High yield	Wild
CIAT_6893	Kenia	Erect	Persistent	Wild
CIAT_6986	Kenia	Erect	High flowering index and in vitro dry matter digestibility	Wild
Mombaça (CIAT_6962)	Korogwe, Tanzania	Erect	Commercial cultivar, high biomass, good drought and cold tolerance	Released cultivar
Naturalizada	Colombia	Erect	Locally adapted, good persistence	Landrace

## Data Availability

The data presented in this study is available on request from the corresponding author. The data is not publicly available due to ongoing analyses and institutional data management policies.

## Supplementary Materials

The following supporting information can be downloaded at https://www.mdpi.com/article/10.3390/plants15172625/s1: MS1: General results of the NCII analysis for agronomic traits; MS2: General results of the NCII analysis for nutritional quality traits. MS3: Complete list of heritability estimators and equations used in the NCII analysis.

## Abbreviations

The following abbreviations are used in this manuscript:

GCA	General combining ability
SCA	Specific combining ability
OCS	Optimal contribution selection
