# Supplementary material for: Combining Ability Analysis and Identification of Testers for Biomass Yield and Forage Quality Traits in Guineagrass (Megathyrsus maximus)
Source: Plants (Basel). 2026 Aug 28;15(17):2625. doi: 10.3390/plants15172625 (PMC13567144; doi:10.3390/plants15172625)
Supplement: Supplementary file 1 [file plants-15-02625-s001.zip › MS3 Heritability estimators and stability ratios used in the NCII analysis.pdf]

Heritability estimators and stability ratios used in the NCII analysis

Estimator	Symbol	Equation	Interpretation	Main reference
Individual narrow-sense heritability from the NCII framework	h_i^2	$h_i^2 = \frac{\sigma_A^2}{\sigma_P^2}$	Proportion of individual phenotypic variance explained by additive genetic variance derived from the NCII framework.	Falconer and Mackay, 1996; Isik et al., 2017; Bernardo, 2020
Female half-sib family-mean narrow-sense heritability	h_{HSF}^2	$h_{HSF}^2 = \frac{\sigma_F^2}{\sigma_{P,HSF}^2}$	Reliability for selecting sexual female parents based on the mean performance of their half-sib families.	Comstock and Robinson, 1952; Falconer and Mackay, 1996; Isik et al., 2017
Male half-sib family-mean narrow-sense heritability	h_{HSM}^2	$h_{HSM}^2 = \frac{\sigma_M^2}{\sigma_{P,HSM}^2}$	Reliability for selecting apomictic male parents or testers based on the mean performance of their half-sib families.	Comstock and Robinson, 1952; Falconer and Mackay, 1996; Isik et al., 2017
Pooled half-sib family-mean narrow-sense heritability	h_{HSP}^2	$h_{HSP}^2 = \frac{0.5(\sigma_F^2 + \sigma_M^2)}{\sigma_{P,HSP}^2}$	Combined reliability of parental selection using both female and male half-sib information.	Isik et al., 2017; Bernardo, 2020
Full-sib family-mean narrow-sense heritability	h_{FS}^2	$h_{FS}^2 = \frac{\sigma_M^2 + \sigma_F^2}{\sigma_{P,FS}^2}$	Reliability of selection among full-sib family means based only on average parental effects, that is, GCA components.	Falconer and Mackay, 1996; Bernardo, 2020
Full-sib family-mean broad-sense heritability	H_{FS}^2	$H_{FS}^2 = \frac{\sigma_M^2 + \sigma_F^2 + \sigma_{MF}^2}{\sigma_{P,FS}^2}$	Reliability of selection among full-sib family means based on total family genetic effects, including GCA and SCA.	Falconer and Mackay, 1996; Bernardo, 2020
Father GCA stability ratio across locations	S_M	$S_M = \frac{\sigma_M^2}{\sigma_M^2 + \sigma_{LM}^2}$	Proportion of father GCA variance expressed as a main effect rather than as father GCA × location.	Grieder et al., 2012; Singh et al., 2014
Female-parent effect stability ratio across locations	S_F	$S_F = \frac{\sigma_F^2}{\sigma_F^2 + \sigma_{LF}^2}$	Proportion of female-parent effect variance expressed as a main effect rather than as female-parent effect × location.	Grieder et al., 2012; Singh et al., 2014

SCA stability ratio across locations	S_{MF}	$S_{MF} = \frac{\sigma^2_{MF}}{\sigma^2_{MF} + \sigma^2_{LMF}}$	Proportion of SCA variance expressed as an average specific combining ability effect rather than as SCA × location.	Grieder et al., 2012; Singh et al., 2014

Denominators used in the equations

Denominator	Multi-environment agronomic traits	Single-location nutritional quality traits
$\boldsymbol{\sigma^2_P}$ for $\boldsymbol{h^2_i}$	$\sigma^2_F + \sigma^2_M + \sigma^2_{MF} + \sigma^2_{LF} + \sigma^2_{LM} + \sigma^2_{LMF} + \sigma^2_e$	$\sigma^2_A + \sigma^2_D + \sigma^2_{design} + \sigma^2_e$
$\boldsymbol{\sigma^2_{P,HSF}}$	$\sigma^2_F + \frac{\sigma^2_M}{m} + \frac{\sigma^2_{MF}}{m} + \frac{\sigma^2_{LF}}{n_l} + \frac{\sigma^2_{LM}}{n_l m} + \frac{\sigma^2_{LMF}}{n_l m} + \frac{\sigma^2_e}{n_l m r}$	$\sigma^2_F + \frac{\sigma^2_M}{m} + \frac{\sigma^2_{MF}}{m} + \frac{\sigma^2_e}{mr}$
$\boldsymbol{\sigma^2_{P,HSM}}$	$\frac{\sigma^2_F}{f} + \sigma^2_M + \frac{\sigma^2_{MF}}{f} + \frac{\sigma^2_{LF}}{n_l f} + \frac{\sigma^2_{LM}}{n_l} + \frac{\sigma^2_{LMF}}{n_l f} + \frac{\sigma^2_e}{n_l f r}$	$\frac{\sigma^2_F}{f} + \sigma^2_M + \frac{\sigma^2_{MF}}{f} + \frac{\sigma^2_e}{fr}$
$\boldsymbol{\sigma^2_{P,HSP}}$	$\frac{\sigma^2_{P,HSF} + \sigma^2_{P,HSM}}{2}$	$\frac{\sigma^2_{P,HSF} + \sigma^2_{P,HSM}}{2}$
$\boldsymbol{\sigma^2_{P,FS}}$	$\sigma^2_M + \sigma^2_F + \sigma^2_{MF} + \frac{\sigma^2_{LM} + \sigma^2_{LF} + \sigma^2_{LMF}}{n_l} + \frac{\sigma^2_e}{n_l r}$	$\sigma^2_M + \sigma^2_F + \sigma^2_{MF} + \frac{\sigma^2_e}{r}$

Notes:

$$\sigma^2_M$$

father GCA variance.

$$\sigma^2_F$$

female-parent average effect variance.

$$\sigma_{MF}^2$$

male × female interaction variance, interpreted as SCA.

$$\sigma_{LM}^2$$

father GCA × location variance.

$$\sigma_{LF}^2$$

female-parent effect × location variance.

$$\sigma_{LMF}^2$$

SCA × location variance.

$$\sigma_e^2$$

average residual variance.

$$n_l$$

number of locations.

$$r$$

average number of observations per family.

$$m$$

average number of male parents per female parent.

$$f$$

average number of female parents per male parent.

For the nutritional quality traits, σ^2_{design} corresponds to the design-related variance adjusted in the single-location model, mainly row and column within batch.
